# A Comparative Study on the Effects of Mesenchymal Stem Cells and Their Conditioned Medium on Caco-2 Cells as an In Vitro Model for Inflammatory Bowel Disease

**DOI:** 10.1155/2024/1022338

**Published:** 2024-10-21

**Authors:** Naser Kalhor, Hoda Fazaeli, Faezeh Davoodi Asl, Azar Sheikholeslami, Seyed Jalal Eshagh Hoseini, Mohsen Sheykhhasan

**Affiliations:** ^1^Department of Mesenchymal Stem Cells, Academic Center for Education, Culture, and Research (ACECR), Qom Branch, Qom, Iran; ^2^Department of Surgery, Qom University of Medical Science, Qom, Iran

**Keywords:** adipose tissue, bone marrow, inflammatory bowel diseases, menstrual blood, mesenchymal stem cells

## Abstract

Recent research indicates that mesenchymal stem cells (MSCs), known for their anti-inflammatory and anti-infectious properties, could be a promising alternative for treating inflammatory diseases such as inflammatory bowel disease (IBD). This study examined how MSCs and their derivatives, when cocultured with interleukin-1*β* (IL-1*β*)-stimulated Caco-2 cells, affect the expression of genes related to inflammation, microbes, and apoptosis. In the experiment, Caco-2 cells were exposed to 10 ng/mL of IL-1*β* for 24 h. MSCs were sourced from human bone marrow, adipose tissue (AD-MSC), and menstrual blood. These MSCs and their conditioned medium (CM) were then cocultured with the IL-1*β*-induced Caco-2 cells. After 48 h, gene expression levels were analyzed using real-time PCR, and the data were statistically evaluated using *T*-tests, *U*-Mann–Whitney, and Tukey's post hoc analyses. The results indicated that IL-1*β* at 10 ng/mL was the optimal concentration for inducing Caco-2 cells with the highest viability and minimal damage. Among the MSCs tested, AD-MSCs were the most effective in regulating gene expression. Specifically, AD-MSC treatment significantly reduced the mRNA expression of TNF-*α* and IL-1*β*, both of which are crucial in sustaining inflammatory responses (*p* ≤ 0.05). This study concludes that AD-MSCs have superior effects compared to other MSC sources in modulating genes associated with inflammation, antibacterial effects, and apoptosis in an in vitro model of IBD using Caco-2 cells.

## 1. Introduction

Inflammatory bowel disease (IBD), as a set of illnesses characterized by a complex and multifaceted disease, is linked to genetics, environment, intestinal microbes, and mucosal immune responses that are out of balance [1–4]. The risk factors in all of these conditions cause the intestinal epithelial barrier to be destroyed, which leads to increased vascular permeability and causes mucosal immune activation and mucosal tolerance failure [5, 6]. A decreased immune response causes gastrointestinal inflammation, which is the hallmark of this illness [7]. Patients with IBD may experience gastrointestinal pain, diarrhea, hematochezia, and unintentional weight loss at first [7]. Malnutrition, fibrostenotic strictures, the formation of fistulas, intra-abdominal abscesses, and colorectal cancer (CRC) are all serious sequelae of unmanaged IBD [7]. This is most common in the age range of 15–30, and the number of people affected is rising worldwide, particularly in developing nations [8, 9]. The remission of IBD is linked to a variety of methods, including drugs and surgery. IBD is treated with anti-integrin antibodies, antitumor necrosis factor, and immunosuppressive drugs such as methotrexate, 6-mercaptopurine, and azathioprine [9]. However, long-term usage of immunosuppressive drugs has resulted in some side effects [10]. Intestinal epithelial cells, in particular, perform an important role as a protective forefront of immunity of the host mucosa, allowing the host to survive persistent interaction with external triggers such as gram-negative infections [7, 11]. The most frequently used human cell line in studies on the activity and integrity of the barrier around the intestines is Caco-2 [12]. In several IBD investigations, Caco-2 cells were employed as an advanced in vitro triple-culture model [13–15]. However, multiple triggers, including its induction with interleukin-1*β* (IL-1*β*), have been utilized to produce an inflammatory state in order to replicate a physiological situation comparable to that in Caco-2 cells. The classic multifunctional cytokine interleukin-1 (IL-1) contributes significantly to the intestinal inflammation associated with Crohn's disease (CD) and other inflammatory disorders of the gut [16]. Earlier studies using in vivo animal model systems as well as in vitro cell culture model systems have demonstrated that IL-1 increases intestinal epithelial tight junction (TJ) resistance [16, 17]. It has been hypothesized that one significant pathogenic process causing intestinal inflammation is the IL-1-induced elevation of intestinal epithelial TJ permeability [16, 18]. As a result, IL-1*β*-induced Caco-2 cells can be proposed as a cell model of IBD [16, 19]. In cell therapy techniques, stem cells and some resident immune system cells have been extensively used [10].

Mesenchymal stem cells (MSCs) and the conditioned medium derived from MSCs (MSC-CM) have been shown to have therapeutic effects on a variety of inflammatory disorders, including acute kidney injury, pulmonary hypertension, arthritis, experimental autoimmune encephalomyelitis, and diabetes [20–22]. According to the latest studies, secreted paracrine substances may mediate some of the therapeutic actions of MSCs. These paracrine factors, also known as MSC-CM, may decrease apoptosis, encourage angio- and artery formation, and stimulate endogenous healing [23]. Among various sources of MSCs, adipose tissue and bone marrow are the two adult tissues from which MSCs are most frequently obtained [24–31]. Besides, menstrual blood is a novel source of stem cells that can be readily obtained from female volunteers in a noninvasive manner without requiring ethical evaluation [32]. Furthermore, EVs derived from bone marrow, adipose tissue, and menstrual blood show promise as potential therapeutic agents for IBD treatment [26, 33–37].

Numerous studies have demonstrated that after cell or tissue damage, MSCs can be triggered by cytokines associated with inflammation, move to the area of damage, and regulate the tissue-regeneration process by secreting a variety of substances that may promote the growth and development of progenitor cells while reducing inflammatory responses [38]. MSCs can also respond to inflammatory cytokines and interact with adaptive and innate immune components by secreting immunomodulatory particles that influence T cells, dendritic cells, natural killer (NK) cells, macrophages, and B cells, thereby controlling inflammation progression [39]. In accordance with the therapeutic properties of MSCs, numerous clinical trials are being conducted employing MSCs to treat a number of immune-mediated disorders, including CD and multiple sclerosis (MS) [40, 41]. Hence, MSCs have been shown to be a viable therapeutic alternative for the treatment of immune-related illnesses, such as CD, and refractory inflammatory diseases [40]. Furthermore, MSCs could be the best source for treating IBD [42]. MSCs have the ability to engraft the intestinal mucosa, control inflammation, and hence heal damaged tissues [42]. In fact, transplanted MSCs support the effective elimination of harmful bacteria and restore altered gut microbiota [42].

As a result, genes associated with inflammation, antibacterial characteristics, and apoptosis may play an important role in the development of IBD.

IBD's core component is excessive or poorly managed inflammation [43]. Many genes are associated with inflammation. Some of the most significant genes involved in inflammation are caudal-type homeobox 2 (CDX2), tumor necrosis factor-*α* (TNF-*α*), interleukin-12 (IL-12), IL-1*β*, matrix metallopeptidase 9 (MMP9), and monocyte chemoattractant protein-1 (MCP-1). CDX2 controls the homeostasis of the intestine. There is more and more proof that CDX2 causes inflammation in the intestines. Inflammation starts when CDX2 expression is altered. A downstream mediator of cytokines that promote inflammation is CDX2 [44]. An essential part of the etiology of IBD is played by the proinflammatory mediator TNF-*α*. Additionally, accumulating data suggests a genetic link between TNF-*α* and colitis with ulcers [45]. One of the main cytokines responsible for triggering and maintaining intestinal inflammation in IBD is IL-12 [46]. In addition, colon inflammation is first brought on by IL-12 acting on innate immune system cells [47]. A powerful proinflammatory cytokine called IL-1*β* is essential for the host's defense mechanisms in response to infection and damage. Of the 11 members of the IL-1 family, it is also the most researched and best characterized [48]. By luring monocytes and macrophages, MCP-1 plays a significant role in inflammation [49]. It has been proven that IBD causes an increase in MMP9 expression. MMP9 is also a significant tissue damage mediator in colitis [50].

There is growing evidence that gut microbes play a pivotal role in IBD and CRC [51, 52]. According to evidence, NOD1 and NOD2 are interesting therapeutic targets because they produce intracellular bacterial sensor proteins that operate as innate immune stimuli [53].

It was discovered that the intestinal epithelium of people with IBD has a greater rate of apoptosis [54]. In the early phases of ulcerative colitis (UC)–associated colon carcinogenesis, it is thought that the p53 gene mutation plays a significant role, and the expression of proteins of p53 in UC-associated colon cancers may be a useful biomarker [55].

In this study, we investigated whether the anti-inflammatory, antimicrobial, and antiapoptotic properties of MSCs and their conditioned medium can affect Caco-2 cells as the intestinal inflammatory model, with a vision for ameliorating IBD in the future.

## 2. Material and Methods

### 2.1. Chemicals

DMEM/low glucose medium was purchased from Gibco (Grand Island, United States). IL-1*β* was bought from Sigma-Aldrich in St. Louis, Missouri.

### 2.2. Cell Culture

Caco2 was employed from a human colon adenocarcinoma (RRID: CVCL_0025), which was obtained from the Pasteur Institute (Tehran, Iran) and cultivated in DMEM with 10% (*v*/*v*) fetal bovine serum (FBS) (Gibco, Grand Island, United States) and 1% (*v*/*v*) penicillin/streptomycin (Pen/Strep) (Gibco, Grand Island, United States). A 5% CO_2_/95% humidity (*v* : *v*) environment was used to culture the cells in 175 cm^2^ flasks (Greiner, Bio-One, Strickenhausen, DE) at a temperature of 37°C.

### 2.3. Isolation and Culture of MSCs From Menstrual Blood

This interventional experimental study was confirmed by the Research Ethics Committees of the Islamic Azad University-Qom Branch (IR.IAU.QOM.REC.1399.049). Before participating in the study, three volunteers signed a written informed consent form. During the second or third day of menstruation, menstrual blood was taken using a pipelle catheter and immediately sent to the laboratory. Mononuclear cells were separated using Ficoll-Paque (Lymphodex, Innotrain, Germany) and density-gradient centrifugation as directed by the manufacturer. In brief, after centrifugation (600 g for 30 min), the buffy coat was recovered and cleaned twice with sterile phosphate-buffered saline (PBS) before centrifugation (400 g for 30 min) at room temperature (RT). The pellets were then cultured in DMEM-low glucose medium (Gibco, Grand Island, United States) containing 10% FBS (Gibco, Grand Island, United States) and 1% Pen/Strep (Gibco, Grand Island, United States) at 37°C in a humidified environment with 95% humidity and 5% CO_2_ [56, 57]. When the cells had reached around 80% confluency, trypsinization, subculturing, and cell passage were performed.

### 2.4. Isolation and Culture of MSCs From Adipose Tissue

After receiving informed written consent, adipose tissue samples were obtained from three volunteers who had liposuction surgery. Then, in a sterile laboratory and under the laminar hood, adipose tissue samples were sliced into small pieces with sterile scissors. The adipose sample was minced several times with a PBS solution in order to eliminate blood cells and debris. 1.5 mg/mL Collagenase Type I was used to digest adipose tissue pieces and was kept at 37°C for 45 min in a humidified environment with 97% humidity and 5% CO_2_. Cell suspensions were centrifuged for 8 min at 1800 rpm, then the pellet was resuspended and grown in DMEM with 10% FBS and 1% Pen/Strep. Finally, the flask with the cell was placed in an incubator at 37°C, 5% CO_2_, and 95% humidity [58, 59]. The media was replaced every 3–4 days after stem cell isolation. In Passages 2–4, these cells were employed.

### 2.5. Isolation and Culture of MSCs From Bone Marrow Tissue

After getting informed written consent, an oncologist collected a bone marrow sample from three preferably young persons undergoing leukemia treatment at Khorami Hospital in Qom Province, and it was subsequently transmitted to the laboratory. After that, the aspirated blood sample was given an equivalent volume of PBS. Pour 5 mL of diluted blood from the Falcon wall gently into 3 mL of the follicle, then centrifuge for 25 min at 2000 rpm at 25°C. Separate the mononuclear cells from the other layers and add around 9 mL of growth media. After that, it was centrifuged at 1300 rpm for 10 min at 25°C. Approximately 1 mL of the cell suspension is equally cultured in 4 mL of culture medium (including 10% serum [FBS in a T25 flask]) and placed in an incubator (37°C and 5% CO_2_). When the cells have adhered to the bottom of the container, discard the culture medium on the cells' surface and slowly replace it with the same amount of fresh culture medium. After washing the cells with a PBS wash solution, the culture medium was replaced [58]. Cell passaging occurs 14 days after the start of the initial culture when the cells have reached a density of 70%–80%. Remove the cells from the growing medium and wash them with the washing solution. Trypsin is put on the surface of the cells, and the cells are collected and moved to two or more new flasks containing fresh culture medium, which are utilized in Passages 2–4 after a brief period (2–5 min) at 37°C by removing the cells from the bottom of the container.

### 2.6. Confirmation of MSC Marker Expression

MSCs were differentiated into three lineages: cartilage, bone, and adipose tissue. According to three of our previous studies, AD-MSC, BM-MSC, and MB-MSC were isolated and cultured using the same methodology, and cells were verified by inverted microscope for morphology and real-time PCR for triple differentiation ability into chondrocyte, osteocyte, and adipocyte lineages. Flow cytometry tests were also done on AD-MSC, BM-MSC, and MB-MSC for the evaluation of CD73 and CD90 as positive markers and CD34 and CD45 as negative markers, according to our earlier work [57–60]. Briefly, MSCs in the third passage were labeled with antihuman antibodies, including anti-CD73, anti-CD90, anti-CD34, and anti-CD45 antibodies, for flow cytometric analysis. A FACSCalibur flow cytometer was used to perform the analysis.

MSCs were trilineage-differentiated into osteocytes, adipocytes, and chondrocytes, according to our previous studies [27, 58, 59]. To make a chondrogenic medium, combine DMEM High Glucose with 1% insulin–transferrin–selenium, 100 nM dexamethasone, 1% human serum albumin (HSA), 50 g/L ascorbate, 5 g/L linoleic acid, TG Growth Factor 3, and 1% Pen/Strep. Finally, the expression of aggrecan and Collagen Type II genes is assessed to determine whether MSCs can differentiate into the cartilage lineage.

The bone medium is made up of DMEM High Glucose with 2 mM sodium phosphate, 100 nM dexamethasone, 50 mg/L ascorbate, 10 mM beta-glycerol phosphate, and 1% Pen/Strep. Finally, the expression of the alkaline phosphatase gene was tested to validate MSC differentiation into bone lineages. The adipocyte-inducing differentiation medium was made up of DMEM High Glucose with 0.1 mg/mL insulin, 1 M dexamethasone, 1 mM 3-isobutyl-1-methylxanthine, 0.2 mM indomethacin, and 1% Pen/Strep. Finally, the expression of the PPAR gene was assessed in order to confirm MSC differentiation into the adipocyte lineage [27].

### 2.7. Preparation of MSC-CM

In the third passage, 20,000 MSCs per square centimeter were cultured and incubated in DMEM/low glucose medium (Gibco, Grand Island, United States) containing 10% FBS (Gibco, Grand Island, United States) and 1% Pen/Strep (Gibco, Grand Island, United States) for 24 h at 37°C in a humidified environment with 5% CO_2_. The seeded cells were rinsed multiple times with PBS before being cultured for 2 days in the new DMEM+FBS medium+Pen/Strep medium. The MSC-CM was collected. To remove cell debris, the collected MSC-CM was centrifuged at 1800 × g for 10 min. A 0.2 filter was used to filter the MSC-CM that was collected [61]. The collected medium is stored at −80°C.

### 2.8. Coculture MSCs and the Caco-2 Cell Line

For coculture, the Caco-2 cell line was cultured in six-cell cell culture plates at a density of 80,000 cells per well. These cells were exposed to IL-1 beta at a concentration of 10 ng/mL for 24 h. In each well, 30,000 cells from three sources of MSCs (AD-MSC, BM-MSC, and MB-MSC) were separately cocultured with IL-1 beta–treated Caco-2 cells.

### 2.9. Coculture Conditioned Medium From MSCs and the Caco-2 Cell Line

To make the conditioned medium, we culture the MSCs first, then discard the culture medium when they have reached a confluency of 80%–70% and replace it with a new medium. After 48 h, remove the top culture medium, which is prepared as a conditioned medium by centrifugation at 2000 rpm for 5 min. The Caco-2 cell line was grown on six-well cell culture plates with a density of 80,000 cells per well for coculture. IL-1 beta was given to these cells for 24 h at a dosage of 10 ng/mL. In each well, the conditioned medium obtained from the culture of 30,000 cells of three sources of MSCs (AD-MSC, BM-MSC, and MB-MSC) was independently cocultured with Caco-2 cells that had been exposed to IL-1 beta.

### 2.10. Study Design

**Figure 1:**
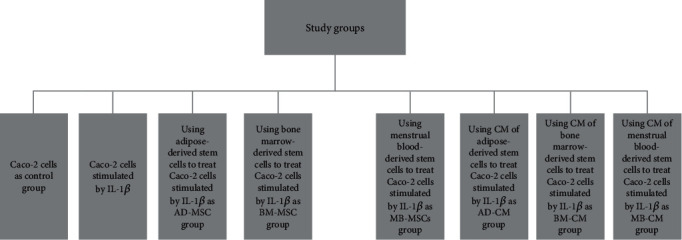
The flowchart of the study design.

The study was designed for eight groups of MSCs and the conditioned medium resulting from them (Figure 1), which were defined as follows:

1. Control group (Caco-2 cells)
2. IL-1*β*-induced Caco-2 cells
3. AD-MSC group (treatment of IL-1*β*-induced Caco-2 cells with adipose-derived stem cells)
4. BM-MSC group (treatment of IL-1*β*-induced Caco-2 cells with bone marrow–derived stem cells)
5. MB-MSC group (treatment of IL-1*β*-induced Caco-2 cells with menstrual blood–derived stem cells)
6. AD-CM group (treatment of IL-1*β*-induced Caco-2 cells with CM of adipose-derived stem cells)
7. BM-CM group (treatment of IL-1*β*-induced Caco-2 cells with CM of bone marrow–derived stem cells)
8. MB-CM group (treatment of IL-1*β*-induced Caco-2 cells with CM of menstrual blood–derived stem cells)

### 2.11. Cell Viability Assay

The MTT assay was used to assess the effects of IL-1*β* on the viability of the Caco-2 cell line. Three times, the MTT assay was carried out (24, 48, and 72 h). Following the incubation durations, 20 *μ*L of MTT solution (5 mg/mL) was placed into each well. The cells were then incubated for the final 4 h at 37°C with 5% CO_2_ in the MTT solution. The formazan crystals were then dissolved in a 100-*μ*L solution of isopropanol hydrochloride. On a microplate reader (Biokit ELx800 Reader, Spain), the optical density (OD) of the formazan was determined at 570 nm wavelength. All of the experiments were done in triplicate.

### 2.12. Evaluation of Gene Expression in Cells by Real-Time qRT-PCR

Total RNA was extracted using GeneAll solution (Gene All Biotechnology, Seoul, Korea), and cDNA was synthesized using the GeneAll kit (Gene All Biotechnology, Seoul, Korea) as directed by the manufacturer. For real-time PCR performance, the Applied Biosystems StepOne Plus (ABi) was also employed. All samples are tested four times.

The GeneAll kit (Gene All Biotechnology, Seoul, Korea) was used to isolate total RNA from treated cells according to the manufacturer's instructions. RNA purity and quantity were assessed using a Nanodrop 2000 spectrophotometer (Thermo Fisher Scientific, Wilmington, United States) at 260/280 nm. Using the transcription kit (Yekta Tajhiz, Iran), reverse transcription was utilized to create the first-strand cDNA. Three-fold duplicate quantitative real-time PCR assays were run to evaluate the expression of the selected genes (Table 1).

The glyceraldehyde-3-phosphate dehydrogenase (GAPDH) gene was utilized as an internal reference for normalizing gene expression levels. The fold change of mRNA expressions for target genes was calculated using the 2^−ΔΔCt^ method. RealQ Plus Master Mix Green (AMPLIQONIII) was used for real-time PCR in accordance with the manufacturer's instructions. To reach a final volume of 20 *μ*L, a combination consisting of 10 *μ*L SYBR green mix, 1 *μ*L cDNA (250 ng), 1 *μ*L PCR forward primers, and 1 *μ*L PCR reverse primers in 5 pmol/*μ*L, and Millipore water was added. The primer sequences are displayed in Table 1. For every run, the threshold cycle (CT) was manually calculated. The relative fold change, or relative mRNA level, was estimated with the formula 2^–ΔΔCT^ = 2^–(ΔCT(Sample) − ΔCT(reference))^; ΔCT = ΔCT Target–ΔCT GAPDH. A calibrator sample was chosen from the control group, which received no treatment. The quantification of mRNA was performed as a value relative to an internal reference for GAPDH.

### 2.13. Statistical Analysis

The data were analyzed using several statistical methods to ensure a robust and comprehensive evaluation. Specifically, the *T*-test was employed for comparing means between two groups, while the *U*-Mann–Whitney test was used for nonparametric comparisons. For comparisons involving more than two groups, analysis of variance (ANOVA) was conducted, followed by Tukey's post hoc test to identify specific group differences. The statistical analysis was performed using SPSS Version 16. A *p* value of 0.05 or less was considered statistically significant, indicating a less than 5% probability that the observed differences occurred by chance. All tests were two-tailed unless otherwise specified.

## 3. Results

### 3.1. Morphology and Characteristics of MSCs

**Figure 2:**
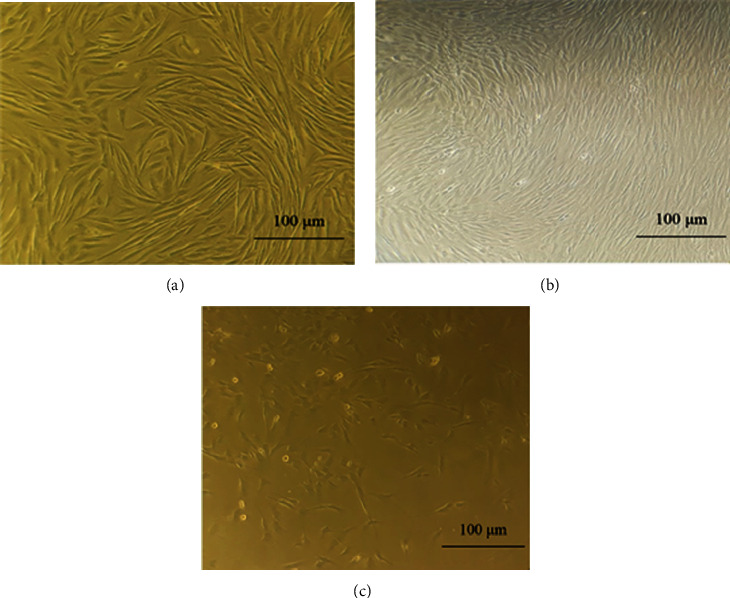
The image produced by phase-contrast microscope of living mesenchymal stem cells isolated from (a) human menstrual blood, (b) bone marrow, and (c) adipose tissue which spindle cells in the third passage are visible.

MSCs were obtained from the adipose tissue, bone marrow, and menstrual blood. The appearance of these cells was similar to that of fibroblast cells (Figure 2). MSCs were able to proliferate to Passage 3 by maintaining morphological characteristics. In our study, microscopic observations showed that MSCs from patients had fibroblast-like, spindle-shaped morphology.

**Figure 3:**
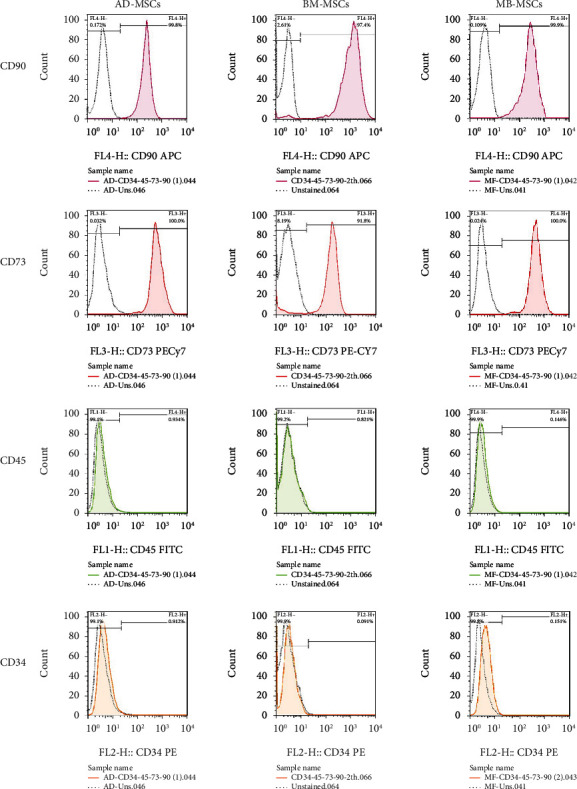
MB-MSC, BM-MSC, and AD-MSC surface marker expression, including CD73 and CD90 as positive markers and CD34 and CD45 as negative markers.

A flow cytometry assay was used to characterize MSCs in Passage 3, and the results revealed that MSCs were positive for CD73 and CD90 (MSC-specific markers), but negative for CD34 and CD45 [57] (Figure 3).

**Figure 4:**
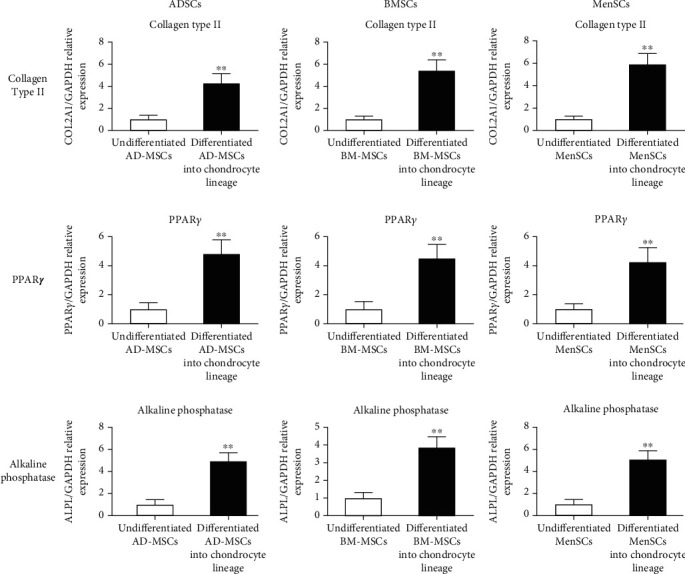
mRNA expression of chondrogenic, adipogenic, and osteogenic markers assessed after 14 days by real-time PCR analysis in mesenchymal stem cells isolated from human adipose tissue, bone marrow, and menstrual blood to confirm their capacity for chondrogenic, adipogenic, and osteogenic differentiation. Note: All data were expressed as mean ± standard deviation. Statistical differences were determined with ANOVA and Duncan's tests, and differences were statistically significant at *p* ≤ 0.5.

The differentiation potential of ADSC into mesodermal lineages such as the chondrogenic, adipogenic, and osteogenic lineages was evaluated using the real-time PCR method to verify multipotent MSCs. The expression levels of the Collagen Type II gene in differentiated MSCs into the chondrocyte lineage were also shown to be considerably different from undifferentiated MSCs (Figure 4). In differentiated MSCs into the adipocyte lineage, relative mRNA levels of the PPAR*γ* were considerably greater (*p* < 05; ANOVA) than in undifferentiated MSCs as a control (Figure 4). In differentiated MSCs into osteocytes, we also measured alkaline phosphatase expression as well as differentiated markers of adult osteocyte phenotype. In comparison to undifferentiated MSCs, alkaline phosphatase expression levels in differentiated MSCs into the osteocyte lineage were considerably higher (Figure 4).

### 3.2. Measuring Cellular Proliferation

**Figure 5:**
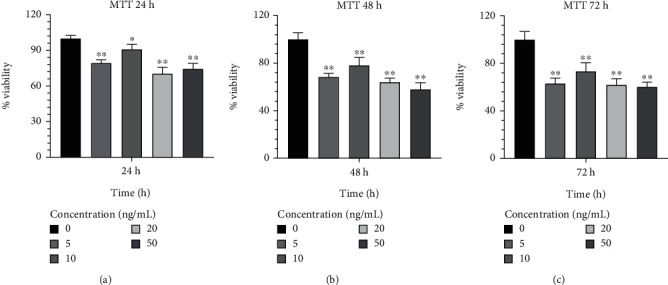
Analysis of cell growth and viability MTT assays were used to evaluate of the viability of IL-1*β* on the Caco-2 cell line. Cells were treated with various concentrations of IL-1*β* at (a) 24, (b) 48, and (c) 72 h. Data were presented as the mean ± standard deviation (*n* = 5). ⁣^∗^*p* < 0.05 versus control and ⁣^∗∗^*p* < 0.01 versus control.

In order to determine the optimal concentrations of IL-1*β* that are not harmful to cells, Caco-2 cells were treated with a range of concentrations from 5 to 50 ng/mL. In comparison to other concentrations, 10 ng/mL of IL-1*β* exhibited the highest viability rates and the least harmful effects on Caco-2 cells during a 24-h incubation period (90.914.18; *p* < 0.05) (Figure 5(a)). Additionally, Caco-2 cells cultured in a medium supplemented with IL-1*β* at 10 ng/mL showed a lower proportion of cell harming after 48 h of growth than other concentrations (78.186.59; *p* < 0.01) (Figure 5(b)). Furthermore, it was discovered that 10 ng/mL of IL-1*β* had considerably greater cell survival and lower detrimental effects than other concentrations after 72 h (73.397.17; *p* < 0.01) (Figure 5(c)) and was proven to be effective. The result from effective concentrations of IL-1*β* suggested the use of the Caco-2 cell line at 10 ng/mL of IL-1*β* for further investigation.

### 3.3. Evaluating the Expression Level of Inflammation, Infection, and Apoptosis-Related Genes

In the present study, we investigated the expression level of inflammation-related genes, including CDX2, TNF-*α*, IL-12, IL-1*β*, MMP9, and MCP-1, in all seven experimental groups in comparison to the control group. In addition, all seven experimental groups were compared to the control group in terms of genes related to infection and inflammation, such as NOD1 and NOD2. In addition, all groups were compared to the control for the p53 gene, which is an important gene connected to apoptosis. In order to analyze the efficacy of the treated and untreated groups, they were also statistically compared. LinRegPCR software was used to investigate the effectiveness of the primers and PCR testing.

### 3.4. Evaluating the CDX2 Expression Level

**Figure 6:**
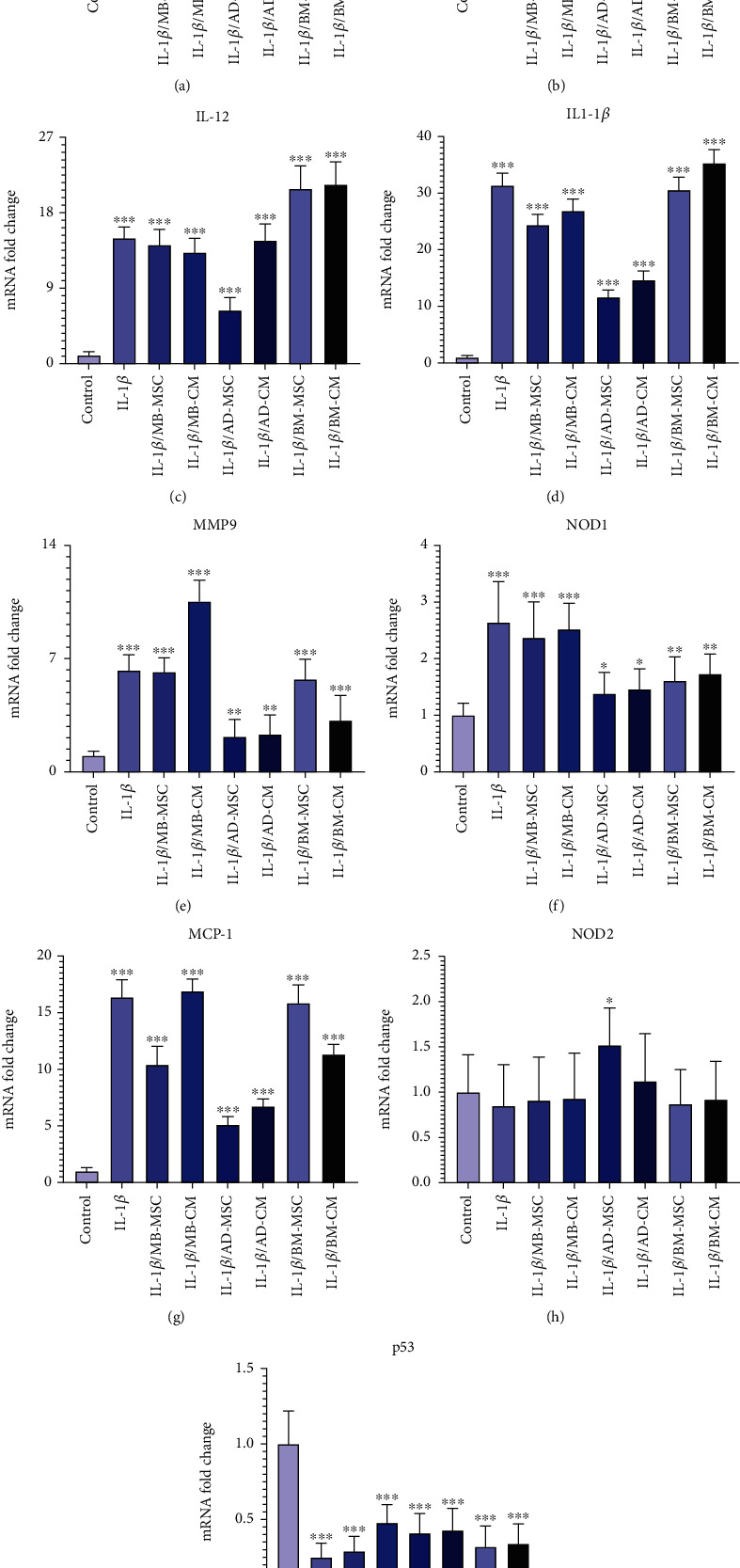
The evaluation of the gene level expression in groups treated with AD-MSCs, BM-MSCs, and MB-MSCs or their CM in comparison to the control group. (a) CDX2, (b) TNF-*α*, (c) IL-12, (d) IL-1*β*, (e) MMP9, (f) NOD1, (g) MCP-1, (h) NOD2, and (i) p53. Statistical differences were statistically significant at *p* ≤ 0.05.

When compared to the control group, real-time PCR revealed significantly lower levels of CDX2 expression in five treated groups except for the AD-MSC group (Figure 6(a)). However, when comparing the CDX2 gene expression levels of the AD-MSC group to the control group, the CDX2 gene expression level of the AD-MSC group was increased (0.98 ± 0.24; *p* = 0.974). The level of CDX2 gene expression in the IL-1*β* group has also decreased significantly compared to the control group (0.19 ± 0.014; *p* < 0.001). In addition, the CDX2 gene expression was considerably higher in the AD-MSC and control groups than in the other groups. Furthermore, the CDX2 gene expression level in the AD-CM group was significantly lower than in the control group (0.45 ± 0.117; *p* < 0.01). Also, BM-MSC caused the lower expression of the CDX2 gene compared to the control group (0.41 ± 0.085; *p* < 0.01). According to our findings, CDX2 expression is considerably lower in the BM-CM group than in the control group (0.32 ± 0.085; *p* < 0.001). The level of CDX2 gene expression in the MB-MSC group has decreased significantly compared to the control group (0.23 ± 0.016; *p* < 0.001). Additionally, MB-CM also had significantly lower CDX2 gene expression than the control group (0.29 ± 0.018; *p* < 0.001).

### 3.5. Evaluating the TNF-*α* Expression Level

When compared to the control group, TNF-*α* gene expression in the IL-1*β* group has dramatically increased (3.652 ± 0.785; *p* < 0.001). The AD-MSC group showed significantly higher relative TNF-*α* expression in coculture with IL-1*β*-induced Caco-2 cells compared with the control group (1.76 ± 0.314; *p* < 0.01), while the expression of the AD-CM group is not significant when compared to the control group; however, the TNF-*α* gene expression level of the AD-CM group compared with the control group was increased (1.385 ± 0.289; *p* = 0.08) (Figure 6(b)). When comparing the TNF-*α* gene expression levels of the MB-MSC group to the control group, the TNF-*α* gene expression level of the MB-MSC group was found to be greater (2.532 ± 0.458; *p* < 0.001). Furthermore, comparing the MB-CM group to the control group revealed a statistically significant increase in TNF-*α* gene expression (3.58 ± 0.602; *p* < 0.001). It was observed that the expression of the TNF-*α* gene has been significantly enhanced in the BM-MSC group compared with the control (4.014 ± 0.853; *p* < 0.001). Additionally, a significant and remarkable increased expression was observed in the BM-CM compared with the control group (3.51 ± 0.347; *p* < 0.001).

### 3.6. Evaluating the IL-12 Expression Level

The IL-12 gene expression in the MB-MSC (14.16 ± 1.865; *p* < 0.001) and MB-CM (13.27 ± 1.678; *p* < 0.001) groups has dramatically enhanced in comparison to the control group. Additionally, when the IL-12 gene's expression in the AD-MSC and AD-CM groups was compared to that of the control, it was shown that there was a considerably higher level of gene expression in the AD-MSC (6.36 ± 1.523; *p* < 0.001) and AD-CM (14.69 ± 1.966; *p* < 0.001) groups (Figure 6(c)). Additionally, it was shown that IL-12 gene expression was significantly higher in the BM-MSC (20.85 ± 2.756; *p* < 0.001) and BM-CM (21.36 ± 2.695; *p* < 0.001) groups than in the control group.

### 3.7. Evaluating the IL-1*β* Expression Level

As shown in Figure 6, in comparison to the control group, the level of the IL-1*β* gene expression has dramatically increased in the IL-1*β* group (31.35 ± 2.146; *p* < 0.001). Compared to the control group, the IL-1*β* gene expression in the MB-MSC (24.36 ± 1.89; *p* < 0.001) and MB-CM (26.87 ± 2.07; *p* < 0.001) groups has also significantly increased. Additionally, it was discovered that the expression of the IL-1*β* gene was significantly higher in the AD-MSC (11.63 ± 1.219; *p* < 0.001) and AD-CM (14.67 ± 1.563; *p* < 0.001) groups than in the control. The IL-1*β* gene expression was also demonstrated to be considerably higher in the BM-MSC (30.56 ± 2.276; *p* < 0.001) and BM-CM (35.31 ± 2.372; *p* < 0.001) groups than in the control group.

### 3.8. Evaluating the MMP9 Expression Level

As seen in Figure 6, the MMP9 group's level of IL-1*β* gene expression has drastically increased when compared to the control group (6.28 ± 0.968; *p* < 0.001). Furthermore, MMP9 expression was significantly reduced not only in the AD-MSC group but also in the AD-CM group in comparison to the other five groups, while there was a significant difference in comparison to the control group (Figure 6(e)). The MMP9 gene expression in the MB-MSC (6.17 ± 0.879; *p* < 0.001) and MB-CM (10.53 ± 1.308; *p* < 0.001) groups has dramatically increased as compared to the control group. Additionally, it was found that when the expression of the MMP9 gene was investigated in the AD-MSC (2.18 ± 1.053; *p* < 0.01) and AD-CM (2.31 ± 1.198; *p* < 0.01) groups, there was a considerably higher level of gene expression compared to the control. Additionally, it was shown that MMP9 gene expression was significantly increased in the BM-MSC (5.73 ± 1.239; *p* < 0.001) and BM-CM (3.18 ± 1.536; *p* < 0.001) groups compared to the control group.

### 3.9. Evaluating the NOD1 Expression Level

When compared to the control group, the NOD1 group's level of IL-1*β* gene expression has significantly increased (2.64 ± 0.719; *p* < 0.001), as shown in Figure 6. NOD1 expression was found to be considerably greater in all groups when compared to the control group (Figure 6(f)). The expression of the NOD1 gene was, however, significantly lower in the AD-MSC and AD-CM groups than in the other groups. As a result, the AD-MSC group was found to have a higher level of expression of the NOD1 gene than the control group (1.38 ± 0.376; *p* < 0.05). Furthermore, as compared to the control group, the expression level of the NOD1 gene was considerably higher in the AD-CM group (1.46 ± 0.358; *p* < 0.05). Also, BM-MSC caused a higher expression of the NOD1 gene compared to the control group (1.61 ± 0.419; *p* < 0.01). According to our present results, the NOD1 expression in the BM-CM group was considerably higher than in the control group (1.73 ± 0.351; *p* < 0.001). Additionally, NOD1 gene expression increased significantly in the MB-MSC (2.37 ± 0.632; *p* < 0.001) and MB-CM (2.52 ± 0.459; *p* < 0.001) groups compared to the control group.

### 3.10. Evaluating the MCP-1 Expression Level

According to Figure 6, the level of MCP-1 gene expression has significantly risen in the IL-1*β* group compared to the control group (16.38 ± 1.537; *p* < 0.001). The MCP-1 gene expression was likewise considerably higher in the MB-MSC (10.41 ± 1.632; *p* < 0.001) and MB-CM (16.91 ± 1.056; *p* < 0.001) groups compared to the control group. Additionally, it was shown that the AD-MSC (5.12 ± 0.706; *p* < 0.001) and AD-CM (6.74 ± 0.637; *p* < 0.001) groups had significantly increased MCP-1 gene expression than the control. Additionally, it was found that BM-MSC (15.85 ± 1.603; *p* < 0.001) and BM-CM (11.34 ± 0.865; *p* < 0.001) groups had significantly increased MCP-1 gene expression than the control group.

### 3.11. Evaluating the NOD2 Expression Level

The mRNA expression of the NOD2 gene was lower in the IL-1*β* group than in the control group. However, it was not statistically different from the control group (0.85 ± 0.435; *p* = 0.705) (Figure 6(h)). Comparing the MB-MSC group to the control group, the expression level of the NOD2 gene has decreased; however, this decrease is not statistically significant (0.91 ± 0.478; *p* = 0.893) (Figure 6(h)). Furthermore, the NOD2 gene's expression level diminished when comparing the MB-CM group to the control group, although this difference is not statistically significant (0.93 ± 0.501; *p* = 0.903). Additionally, the NOD2 gene expression level was not statistically different from the control group while being greater in the BM-MSC (0.87 ± 0.378; *p* = 0.618), BM-CM (0.92 ± 0.419; *p* = 0.837), and AD-CM (1.12 ± 0.527; *p* = 0.419) groups than in the control. Furthermore, the AD-MSC group's NOD2 gene expression level was significantly higher than that of the control group (1.52 ± 0.409; *p* = 0.02).

### 3.12. Evaluating the p53 Expression Level

Real-time PCR demonstrated that the IL-1*β* group had lower levels of p53 expression than the control group (0.25 ± 0.093; *p* <0.001) (Figure 6(i)). p53 gene expression level comparisons between the MB-MSC (0.29 ± 0.098; *p* < 0.001) and MB-CM (0.48 ± 0.119; *p* < 0.001) groups and control participants revealed significant differences. Additionally, p53 gene expression levels in the AD-MSC (0.41 ± 0.129; *p* < 0.001) and AD-CM (0.43 ± 0.143; *p* < 0.001) groups were considerably lower than in the control group. Furthermore, real-time PCR demonstrated that the BM-MSC (0.32 ± 0.137; *p* < 0.001) and MB-CM (0.48 ± 0.119; *p* < 0.001) groups had lower levels of p53 expression than the control group.

## 4. Discussion

Stem cell–based therapeutics have recently been investigated in a variety of immune-mediated inflammatory disorders (IMIDs), including IBD [62]. Since MSCs influence both innate and adaptive immune cells, they have a wide range of immunomodulatory potential. Numerous studies have demonstrated that MSCs limit the proliferation of T lymphocytes that are activated by various polyclonal mitogens or particular antigens [63]. Its inhibitory activity is thought to be caused by either cell cycle arrest in the G0/G1 phase or apoptosis [64]. Furthermore, MSCs alter the cytokine milieu produced by various T cell subsets by producing more anti-inflammatory cytokines and less proinflammatory cytokines [65, 66]. According to previously published research, MSCs have the ability to prevent the proliferation of in vitro activated B cells and their ability to produce immunoglobin [67]. MSCs also have an impact on innate immunity in addition to adaptive immunity. It has been documented that based on the origin, MSC characteristics, especially in terms of differentiation potential and immune regulation, demonstrate a certain extent of differences [68]. In this regard, Wegmeyer et al. examined the biological differences between MSCs from perinatal tissues (umbilical cord and amniotic membrane) and adult BM-MSC [69]. They found that although all MSCs display the same surface markers, the gene expression profile and secreted paracrine factors were different, leading to the higher potential for immunomodulation in MSCs from perinatal tissues versus the higher regenerative potential of BM-MSC. Moreover, when BM-MSC and MB-MSC were isolated from the same donor, about 700 genes that were mostly involved in cell function have been expressed differentially [68]. Therefore, the origin of MSCs can be a contributing factor in the variable outcomes of clinical trials.

Till now, some promising clinical trials using MSCs have been done on IBD patients [70]. The majority of the clinical trials of IBD are performed using AD- and BM-MSC as the two most widely used types of MSCs [71–73]. However, there is a lack of studies comparing the effect of different sources of MSCs on the treatment of IBD. So, in the present study, the effect of three sources of MSCs, including adipose tissue, bone marrow, and menstrual blood, and their conditioned mediums on the gene expression level of IL-1*β*-induced Caco-2 cells as an IBD model was investigated. Moreover, since there is a growing body of evidence that transplanted MSCs' therapeutic and immune-regulatory effects are mostly dependent on the paracrine rather than cellular mechanisms [74, 75], and also due to variable secretions of MSCs based on the tissue of origin [76], the effect of all the MSCs' corresponding conditioned media was also assessed.

It has been demonstrated that IL-1*β* and TNF-*α* cause impairments in intestinal epithelial TJs, leading to increased intestinal permeability [77]. Also, IL-1*β* can cause epithelial cell apoptosis, resulting in tissue damage and barrier disruption [78]. Our results showed that treatment with MB-MSC, AD-MSC, and AD-CM decreased the expression level of IL-1*β* in comparison with the IL-1*β*-induced Caco-2 group (Figure 6(d)). These data are in line with a study by Qi et al. who compare the therapeutic effects of the AD-MSCs–conditioned media and activated conditioned media (collected from pretreated AD-MSCs with the serum from colitis rats) (CM-AcMSC) on dextran sodium sulfate (DSS)–induced colitis rat models. They found that TNF-*α*, IL-1*β*, and IL-6 cytokine mRNA levels were decreased in treated groups with CM-MSC or CM-AcMSC compared to the DSS group, according to RT-qPCR data [79].

CDX2 is a key regulator of homeostasis in the intestinal epithelium, controlling a wide range of cell functions such as proliferation, differentiation, adhesion, and migration. In addition to these critical physiological processes, there is growing evidence that CDX2 is linked to intestinal inflammation (e.g., engagement in proinflammatory pathways and regulation of genes important for intestinal homeostasis) [80–82]. It has been shown that CDX2 is significantly downregulated in intestinal epithelial cell biopsies of patients with active UC [83]. Moreover, the expression pattern of CDX2 was found to be inversely related to that of TNF-*α* [83] which is upregulated in patients with active CD to play a role in the pathogenic processes. All these data are compatible with our results shown in Figures 6(a) and 6(b), whereas in comparison to the control group, a significant down- and upregulation of CDX2 and TNF-*α* in the IL-1*β*-induced Caco-2 group was observed, respectively. In addition, we found that in terms of increasing CDX2 expression, AD-MSC-, AD-CM-, and BM-MSC-treated groups were efficient while the other treated groups were not so. Almost the same, the expression level of TNF-*α* in AD-MSC- and AD-CM-treated groups was decreased with superiority for the AD-CM-treated group, in which the TNF-*α* expression level was more similar to the control group.

The other proinflammatory cytokine upregulated by antigen-presenting cells in the intestinal tissue of CD patients is IL-12, which activates Type 1 helper T (Th1) cells to mediate the inflammatory response [84–86]. In addition, Chapuy et al. recently found that CD163-monocyte-like cells boosted the number of IL-8+IL-17±IFN*γ*±T cells in the inflamed mucosa of UC patients by expressing IL-1*β* and IL-12 [87]. Similarly, our data showed a significant increase in the mRNA level of IL-12 in the IL-1*β*-induced Caco-2 group as the IBD model in comparison to the control group. Moreover, as shown in Figure 6(c), the BM-MSC and BM-CM groups showed higher IL-12 expression than all the other groups compared to the IL-1*β*-induced Caco-2 group. In the AD-MSC group, the expression of IL-12 was decreased compared to the IL-1*β*-induced Caco-2 group, while it was significantly increased compared to the control group.

MMPs have been shown to be induced in a variety of IBD pathological circumstances and to play a vital role in IBD pathophysiology regulation [88]. Cell migration and cytokine stimulation are aided by active MMP2 and MMP9. Also, the upregulation of MMP9 has been demonstrated to cause neutrophil infiltration in the inflamed intestine of mice [89]. Our data showed a significant elevation in the MMP9 expression level of the IL-1*β*-induced Caco-2 group compared to the control group as well. In addition, in the case of treating MSCs and their CM, MMP9 was lower expressed in the AD-MSC and AD-CM groups in comparison to the IL-1*β*-induced Caco-2 group (Figure 6(d)). In a previous study, it was shown that the clinical and histological indications of DSS-induced colitis in mice were successfully alleviated by systemic infusion of umbilical cord MSCs (UCMSCs), whereas MMP2 and MMP9 activities were increased in DSS-treated animals but were considerably reduced in mice receiving UCMSC [90].

Recent researches on the human microbiota have revealed that the intestinal bacterial community plays a significant role in the pathogenesis of both systemic and intestinal disorders, including CD [91]. NOD1 and NOD2, as two members of the NBS/LRR family of proteins, are suggested to be cytoplasmic “sensors” of microbial products [92]. Regarding NOD1, previous research has highlighted its importance in innate immune responses to bacterial pathogens. NOD1 recognizes bacterial peptidoglycan fragments, particularly those from gram-negative bacteria, triggering intracellular signaling pathways that lead to the production of proinflammatory cytokines and antimicrobial peptides [93, 94]. Dysregulation of NOD1 signaling has been implicated in various inflammatory and autoimmune conditions, including CD [95–97]. Although not directly addressed in our study, the higher expression of NOD1 in the IL-1*β*-induced Caco-2 group compared to the control group suggests its involvement in the inflammatory response observed in our experimental model. Furthermore, the significant decrease in NOD1 expression observed in the treated groups, albeit still higher than the control, indicates the potential of MSCs and their conditioned media to modulate NOD1-mediated inflammatory pathways, which warrants further investigation in the context of CD pathogenesis. Furthermore, it has been shown that loss of function mutations in NOD2 is linked to ileal CD, according to genome mapping of CD patients [91]. In a previous study, TNF-*α*-induced upregulation of NOD2 mRNA in FHC cells of intestinal epithelial origin was discovered [98]. Therefore, the upregulation of NOD2 expression in CD patients may be a generic reaction to bacterial invasion and a futile attempt to reestablish the damaged epithelial barrier. Our data showed that NOD1 was expressed significantly higher in the IL-1*β*-induced Caco-2 group compared to the control group. In addition, although the expression level of NOD1 in AD-MSC-, AD-CM-, BM-MSC-, and BM-CM-treated groups was significantly lower than the IL-1*β*-induced Caco-2 group, it was still significantly higher than the control group. In the case of NOD2 mRNA level, except for the AD-MSC group, which showed a significant increase, there were no significant differences among treated groups and the IL-1*β*-induced Caco-2 group (Figures 6(f) and 6(h)).

Also, it has been shown that MCP-1 expression is increased in IBD, which recruits monocytes to the mucosal lesion [99]. In another study that also demonstrated MCP-1's role in intestinal inflammation, the serum level of MCP-1 was shown to be higher in IBD patients compared to healthy controls [100]. Accordingly, in the present study, the expression level of MCP-1 in the IL-1*β*-induced Caco-2 cell group was significantly elevated compared to the control group. This result is also in line with the previous findings in which the level of MCP-1 mRNA of Caco-2 cells was upregulated by IL-1*β*, while dexamethasone caused its downregulation [101]. Also, in MB-MSC, AD-MSC, BM-CM, and AD-CM, the MCP-1 level was lower than the IL-1*β*-induced Caco-2 cell group (Figure 6(g)). The possible mechanism of decreasing MCP-1 expression level in the mentioned group is weakening the IL-23/Th-17 axis-orchestrated intestinal inflammation and immune impairment. It has been reported that T-cell priming is enhanced by IL-17, which stimulates the expression of proinflammatory cytokines (such as IL-1, IL-6, and TNF-*α*) and chemokines (such as chemokine ligand 1, MCP-1, and macrophage inflammatory protein-2) (23,44), and human UCMSC, in turn, causes downregulation of IL-23 and IL-17 in vivo and in vitro [102].

On the other hand, the downregulation of p53 in inflamed tissues and cells may be the most critical factor in the transformation process. Inflammatory cells, cytokines, chemotactic agents, and growth factors that promote proliferation and suppress apoptosis produce reactive oxygen and nitrogen compounds, which cause DNA oxidative damage that may not be repaired due to down-regulation of p53 [103]. Given epigenetic alterations in oncogenes and other tumor suppressor genes, tumor transformation and progression may be produced. Possibly, this mechanism underlies the long-term inflamed tissues like human UC colonic mucosa [104]. We showed a significant downregulation of p53 in the IL-1*β*-induced Caco-2 cell group in comparison to the control group (Figure 6(i)). Recent researchers have found that IL-6 suppresses p53 expression and activity by stimulating ribosomal ribonucleic acid (rRNA) transcription in proliferation factors and promoting mouse double minute 2 homolog (MDM2)–mediated p53 proteasome digestion [105]. IL-6 has been shown to play a key role in experimental colitis, and its high levels are a substantial risk factor for colon cancer and hepatocellular carcinoma in humans [106]. As a result, it is speculated that under pathological conditions like inflammation, stimulating factors of cell proliferation are produced, and so a similar mechanism could be responsible for tumor transformation.

However, our study has several limitations. Firstly, the in vitro nature of our experimental model may not fully recapitulate the complex pathophysiology of IBD in vivo. Additionally, the mechanisms underlying the observed effects of MSCs on gene expression patterns require further elucidation, and the clinical relevance of our findings warrants validation in animal models and clinical trials. Moreover, the variability in MSC characteristics and secretory profiles based on tissue of origin underscores the need for personalized approaches to MSC therapy in IBD. Despite these limitations, our study provides valuable insights into the therapeutic potential of MSCs and their conditioned media in modulating gene expression patterns associated with IBD pathogenesis.

Considering that the paracrine secretions of MSCs from different sources can be highly variable, which in turn results from the difference in the gene expression pattern of these cells [107], it seems that AD-MSC has more effective content in improving gene expression patterns of IL-1*β*-induced Caco-2 cells.

Almost all the above gene expression patterns showed more efficiency for the AD-MSC group in treated IL-1*β*-induced Caco-2 cells.

## Figures and Tables

**Table 1:** Specific primers for target genes.

**Gene**	**Sequence**	**Accession number**	**Product size (bp)**
CDX2	F: CCAAGTGAAAACCAGGACG	NM_001265.6	104
	R: GGATGGTGATGTAGCGACT		
TNF-*α*	F: CCAGGGACCTCTCTCTAATC	NM_000594.4	91
	R: GCTACAACATGGGCTACAGG		
IL-12*β*	F: CCCAGAGCAAGATGTGTCAC	NM_002187.3	177
	R: CTTCTTCAGGGGTGTCACAG		
IL-1*β*	F: CTGCTCTGGGATTCTCTTCA	NM_000576.3	117
	R: GTCATCCTCATTGCCACTGT		
MMP9	F: CCCTGCCAGTTTCCATTCA	NM_004994.3	196
	R: TGAAGGGGAAGACGCACA		
MCP-1	F: CTCATAGCAGCCACCTTCAT	NM_002982.4	141
	R: CTTGCTGCTGGTGATTCTTC		
NOD1	F: CAACGATGAAGTGGCAGAG	NM_006092.4	148
	R: CCCCTTAGCTGTGATCTGA		
NOD2	F: GGTTTCGTCAGCCAGTATG	NM_022162.3	108
	R: CAATCCATTCGCTTTCACC		
p53	F: GTCATCTTCTGTCCCTTCCCA	NM_001276696.3	117
	R: TTGTTGAGGGCAGGGGAGT		

## Data Availability

The data used to support the findings of this study are available from the corresponding author upon request.
